# Genetic Modulation of Transcranial Direct Current Stimulation Effects on Cognition

**DOI:** 10.3389/fnhum.2016.00651

**Published:** 2016-12-22

**Authors:** Ariane Wiegand, Vanessa Nieratschker, Christian Plewnia

**Affiliations:** ^1^Molecular Psychiatry, Department of Psychiatry and Psychotherapy, University of TübingenTübingen, Germany; ^2^Neurophysiology and Interventional Neuropsychiatry, Department of Psychiatry and Psychotherapy, University of TübingenTübingen, Germany

**Keywords:** brain stimulation, cognition, BDNF, COMT, dopamine, neuroplasticity, stimulation genetics, tDCS

## Abstract

High inter-individual variability substantially challenges the explanatory power of studies on the modulation of cognitive functions with transcranial direct current stimulation (tDCS). These differences in responsivity have been linked with a critical state-dependency of stimulation effects. In general, genetic diversity is a decisive biological basis of variations in neuronal network functioning. Therefore, it is most likely that inter-individual variability of tDCS-induced changes in cognitive functions is due to specific interactions between genetically determined network properties and the specific type of stimulation. In this context, predominantly the brain-derived neurotrophic factor (BDNF) Val66Met and the catechol-O-methyltransferase (COMT) Val108/158Met polymorphisms have been investigated. The studies on the interaction between the BDNF Val66Met polymorphism and the effect of brain stimulation indicate a critical but yet heterogeneous interaction. But up to now, data on the interplay between this polymorphism and tDCS on cognitive functioning are not available. However, recently, the functional Val(108/158)Met polymorphism in the COMT gene, that is particularly involved in the regulation of executive functions by means of the dopaminergic tone in frontal brain areas, has been demonstrated to specifically predict the effect of tDCS on cognitive control. Following an inverted U-shaped function, the high dopaminergic activity in Met allele homozygous individuals has been shown to be associated with a reduction of executive functioning by anodal tDCS to the prefrontal cortex. Consistently, Val homozygous individuals with lower dopaminergic tone show a clear reduction of response inhibition with cathodal tDCS. These findings exemplify the notion of a complex but neurophysiologically consistent interaction between genetically determined variations of neuronal activity and tDCS, particularly in the cognitive domain. Consequently, a systematic analysis and consideration of genetic modulators of tDCS effects will be helpful to improve the efficacy of brain stimulation and particularly tDCS in the investigation and treatment of cognitive functions.

## Introduction

Targeted modulation of cortical areas by means of magnetic impulses or electric stimulation can modify brain functioning and the associated cognitive processes (Parkin et al., 2015; Plewnia et al., 2015). Transcranial direct current stimulation (tDCS) is a well-established neurostimulation technique. With using this method, a weak constant current is applied via scalp electrodes causing a subthreshold alteration of the resting membrane potential and, consequently, a modulation of cortical excitability (Nitsche et al., 2008). Typically, anodal stimulation increases excitability, whereas cathodal stimulation decreases it (Nitsche and Paulus, 2000). This transient modulation of neuronal activity with tDCS can induce specific facilitatory or inhibitory behavioral effects, respectively. However, it is important to note that the simple dichotomy of anodal enhancement and cathodal impairment is not always applicable within the complexity of neurocognitive functioning (Jacobson et al., 2012; Schroeder et al., 2016). Moreover, the neuromodulatory effects are critically affected by the current state of the system, that is they depend on the present activity of the stimulated brain region. This state dependency causes tDCS effects to be task- and activity-specific (Miniussi et al., 2013; Zwissler et al., 2014). Although research in tDCS effects gained increased attention over the past two decades, high variability of effects and sometimes even contradictory results are reported (Horvath et al., 2014). In addition to anatomical (Kim et al., 2014) and psychological differences (Sarkar et al., 2014) the genetic makeup (Witte et al., 2012) of an individual has a major contribution to this interindividual variability. Therefore, to approach this question, the following review focuses on current findings on the genetic factors influencing the malleability of cognitive processes by tDCS and gives a brief outlook on the perspectives of genetically informed, individualized brain stimulation research and treatment.

## Tdcs In Cognition

The first experiments involving tDCS were exploring the effects of motor cortex stimulation (e.g., Fuortes, 1954; Hern et al., 1962; Priori et al., 1998; Nitsche and Paulus, 2000) but over the past years, more attention has been paid to the modulation of cognitive processes (Kuo and Nitsche, 2012). Especially executive functions, often associated with dorsolateral prefrontal cortex (dlPFC) activity, are targeted by different study designs. Corresponding functions like planning ability, cognitive flexibility and working memory are essential to establish goal-directed behavior and to cope with daily life challenges. The modification of activity in the dlPFC by anodal stimulation has often been associated with improved cognitive functions, for instance, better working memory performance (Brunoni and Vanderhasselt, 2014), improved cognitive control (Plewnia et al., 2015) and enhanced planning abilities (Dockery et al., 2009). However, some findings are inconsistent with this association (e.g., Marshall et al., 2005) and, in fact, the effects of cathodal stimulation on cognition are even more diverse (Jacobson et al., 2012; Zwissler et al., 2014; Schroeder et al., 2016).

To address this variability, the influence of genetic factors on stimulation effects has already been investigated in several studies (Li et al., 2015). For this purpose, mainly genes with an established role in the regulation of neuroplasticity (Chhabra et al., 2016), particularly the brain-derived neurotrophic factor (BDNF) Val66Met and the catechol-O-methyltransferase (COMT) Val108/158Met polymorphisms have been investigated.

## Tdcs And The Brain-Derived Neurotrophic Factor (Bdnf)

Brain-Derived Neurotrophic Factor belongs to the family of neurotrophins, which promote cell survival and development (Huang and Reichardt, 2001). It is expressed as a precursor peptide, proBDNF, which is proteolytically cleaved to generate the mature protein (Seidah et al., 1996). Binding of BDNF either to the tropomyosin-related kinase (Trk) B receptor or the p75 receptor activates different intracellular signaling cascades (Patapoutian and Reichardt, 2001). It seems to play an important regulatory role in the neurophysiological processes underlying cognitive functions. For instance, hippocampal-dependent learning paradigms rely on BDNF/Trk signaling (Tyler et al., 2002). Furthermore, BDNF has been shown to be involved in synaptic plasticity (Lu, 2003) as well as in long-term potentiation and depression (Aicardi et al., 2004).

There are several single nucleotide polymorphisms in the gene encoding BDNF (Liu et al., 2005). One of them causes a substitution in the prodomain of BDNF at position 66 of valine to methionine (Val66Met), which impacts BDNF expression and secretion (Mallei et al., 2015). In cultured hippocampal neurons it has been shown that viral transfection with the BDNF Met allele causes less depolarization induced secretion than Val allele transfection (Egan et al., 2003). On the behavioral level, this polymorphism has been associated with impaired executive functions (e.g., Hariri et al., 2003). This renders BDNF as an excellent candidate gene having an impact on the effects of brain stimulation (**Table 1**). It has been shown that the BDNF polymorphism interacts with training-dependent increases in the amplitude of motor-evoked potentials and motor map reorganization, as Val66Met individuals show reduced plasticity relative to Val66Val individuals (Kleim et al., 2006). These findings have also been replicated for plasticity-inducing TMS protocols, to which only Val66Val homozygous individuals showed a neuroplastic response (Cheeran et al., 2008). Furthermore, the investigation of this interaction was extended to transcranial random noise stimulation (tRNS) and tDCS. Only for tDCS protocols heterozygous Val66Met allele carriers displayed an enhanced cortical excitability following anodal stimulation and a more pronounced cortical inhibition after cathodal stimulation as measured by motor evoked potentials. For tRNS there was no group difference observed (Antal et al., 2010). A more recent study investigated an interaction of the Val66Met polymorphism and stimulation duration in older adults on the modulating effects of anodal tDCS on motor cortex plasticity. After 20 min but not after 10 min of anodal stimulation Met allele carriers experienced enhanced corticospinal excitability compared to individuals homozygous for the Val allele (Puri et al., 2015). Furthermore, Strube et al. (2015) demonstrated increased facilitatory effects of anodal stimulation on cortical plasticity in patients suffering from schizophrenia as well as in healthy controls for heterozygous compared to Val allele homozygous individuals. In contrast, cathodal stimulation caused reduced cortical inhibition in heterozygous schizophrenia patients but enhanced inhibitory effect in healthy heterozygotes indicating an interaction of interindividual differences. Another animal study showed that anodal tDCS combined with low-frequency direct synaptic stimulation applied to the motor cortex causes long-lasting synaptic potentiation most likely mediated by the BDNF Val66Met polymorphism as the effect was absent in mice with an inhibited TrkB activity, which is influenced by the BDNF Val66Met polymorphism. Specifically, individuals homozygous for the Val allele demonstrated greater motor skill improvement under anodal tDCS than Met allele carriers (Fritsch et al., 2010). Aiming at a prediction of therapeutic tDCS effects, Brunoni et al. (2013) have examined the interaction of two genetic variants, the BDNF Val66Met and the 5-HTTLPR polymorphism, with the antidepressant effect of tDCS. The latter one describes an insertion/deletion of 44 bp, which regulates the activity of the serotonine transporter (5-HTT) and is a potential susceptibility gene for affective disorders (Collier et al., 1996). Interestingly, they did not find an impact of the BDNF genotype but of the 5-HTTLPR polymorphism on the antidepressant response of tDCS. Specifically, there was no effect of tDCS in homozygous short allele carriers, whereas the number of long alleles appeared to correlate with the stimulation effect. In sum, studies on the interaction between the BDNF Val66Met polymorphism and the effect of brain stimulation on neuronal and behavioral functioning indicate a critical but yet heterogeneous interaction with predominant evidence for a reduced susceptibility of the Met allele carrier. However, findings from clinical trials do not provide support for the notion that the BDNF polymorphism is suitable to predict the efficacy of tDCS as a treatment of depression. To our knowledge, evidence for an association between BDNF polymorphisms and tDCS on cognitive functions is not yet available.

**Table 1:** Overview of previous studies investigating the interaction of the common BDNF Val66Met Polymorphism with brain stimulation effects.

BDNF allele	Effect	Stimulation Target	Method	Population	Study
Met carrier	**⇓** Plasticity	Motor cortex	Motor training/TMS	Healthy subjects	Kleim et al., 2006
Met carrier	**⇓** Plasticity	Motor cortex	Repetitive TMS	Healthy subjects	Cheeran et al., 2008
Val homozygous	**⇑** Plasticity	Motor cortex	Repetitive TMS	Healthy subjects	Antal et al., 2010
Met heterozygous	**⇑** Plasticity	Motor cortex	Anodal and cathodal tDCS	Healthy subjects	Antal et al., 2010
Met carrier	**⇑** Plasticity	Motor cortex	Anodal tDCS	Older healthy subjects	Puri et al., 2015
Val homozygous	**⇓** Plasticity	Motor cortex	Anodal tDCS	Healthy subjects/patients with schizophrenia	Strube et al., 2015
	**⇓** Inhibition	Motor cortex	Cathodal tDCS	Patients with schizophrenia	Strube et al., 2015
Met heterozygous	**⇑** Inhibition	Motor cortex	Cathodal tDCS	Healthy subjects	Strube et al., 2015
Val homozygous	**⇑** Plasticity	Motor cortex	motor training/anodal tDCS	Healthy subjects	Fritsch et al., 2010
Val66Met	No effect	Antidepressant response (DLPFC)	Bifrontal stimulation	Patients with depression	Brunoni et al., 2013

## Tdcs And The Catechol-O-Methyltransferase (Comt)

Another gene, discussed to be involved in cognitive processes and potentially influencing stimulation outcome, is the COMT gene. The COMT enzyme plays a critical role in the degradation of catecholamines, e.g., dopamine by transferring a methyl-group of *S*-adenosylmethionine to the 3-hydroxy group of the catechol (Axelrod and Tomchick, 1958). A functional polymorphism at position 108/158 causing an amino acid exchange from valine to methionine (Val108/158Met) impacts the enzyme’s thermostability as well as its activity. The Met allele results in a more thermolabile and less active COMT phenotype (Lotta et al., 1995; Lachman et al., 1996; Chen et al., 2004). Especially, in the prefrontal cortex, where the expression of dopamine transporters is low, the COMT enzyme plays an important role in regulating dopamine levels (Sesack et al., 1998; Käenmäki et al., 2010). This is also reflected in the fact that the Val108/158Met polymorphism is affecting cognitive functions being associated with prefrontal cortex activity. In patients suffering from schizophrenia as well as unaffected siblings and healthy controls it has been demonstrated that the number of Met alleles positively correlates with prefrontal executive functions and working memory performance assessed by the Wisconsin Card Sorting Test (Egan et al., 2001). This might result from the lower dopamine degradation rate caused by the Met allele. Furthermore, they identified the Val allele as a risk factor for schizophrenia. Although many studies replicated these findings, there were also contradictory results and a meta-analysis concluded that the interaction of the COMT Val108/158Met polymorphism with cognitive performance is questionable (Barnett et al., 2008). However several neuroimaging studies linked differences in prefrontal cortex activity to COMT Val108/158Met genotype. Specifically, Met allele carriers show increased prefrontal activity indicating lower cortical efficiency during emotion processing tasks, whereas Val allele carriers exhibit higher prefrontal activity during cognitive processes (Mier et al., 2010). For optimal cognitive functioning a physiological prefrontal dopamine concentration is required (Goldman-Rakic et al., 2000). The inverted-U shape hypothesis describes a non-linear relationship between cognitive performance and dopamine concentrations. Accordingly, both too high as well as too low concentrations of dopamine are associated with suboptimal cognitive processing (Cools and D’Esposito, 2011). In parallel, the tDCS effects also depend on dopaminergic activity. Administration of L-Dopa has been shown to extend the inhibitory effects of cathodal stimulation and invert the excitatory effects of anodal stimulation to inhibition (Kuo et al., 2008). Of note, this modulatory influence of dopamine turned out to be strongly dose-related with both high and low activation of dopamine receptors preventing plasticity induction with tDCS (Monte-Silva et al., 2010; Fresnoza et al., 2014). These findings point toward a non-linear, inverted U-shaped relationship between dopaminergic activity and neuroplastic changes by tDCS.

Therefore, a behaviorally relevant interaction of tDCS effects with the individual COMT Val108/158Met polymorphism, which is regulating the prefrontal dopamine concentration, must be taken into account and might open new options to integrate the individually variable dispositions to tDCS in the planning and interpretation of brain stimulation studies. In the clinical domain, one study investigated the influence of this polymorphism on the antidepressant response in a TMS protocol. Although no effect of the COMT polymorphism was found, the 5-HT1A serotonergic receptor promoter region polymorphism predicted the treatment outcome (Malaguti et al., 2011). In the context of another clinical application, Shivakumar et al. (2015) reported a better reduction of auditory hallucinations in schizophrenic patients by tDCS treatment in COMT Val allele homozygous individuals compared to Met allele carriers.

In healthy subjects, two recent studies have demonstrated a specific interaction of the COMT polymorphism with both anodal as well as cathodal tDCS during cognitive tests (Plewnia et al., 2013; Nieratschker et al., 2015). They investigated executive functioning using a Parametric Go/No-Go (PGNG) task. This task comprises three levels tapping different aspects of executive functioning: sustained attention, response inhibition and set-shifting abilities (Langenecker et al., 2007). In both experiments tDCS (1mA) was applied during task performance and targeted to the left dlPFC. In the first study, an effect of anodal stimulation was only observed when including genotype information of the COMT Val108/158Met polymorphism. Specifically, the stimulation impaired set-shifting abilities indicated a deterioration of cognitive flexibility in homozygous Met allele carriers but not in Val allele carriers. In the three levels of the PGNG task, no baseline differences were found. The tasks measuring sustained attention and response inhibition were not affected by adding anodal stimulation (Plewnia et al., 2013). Correspondingly, in the second experiment an interaction of stimulation and genotype information has been found for cathodal tDCS. This time an interference of stimulation with response inhibition was found for the overall group but including genotype as a between subjects factor showed that this effect was specific to individuals homozygous for the Val allele. These researchers showed a deterioration of response inhibition specifically under cathodal stimulation (Nieratschker et al., 2015). These complementary studies clearly indicate the decisive influence of the individual genetic profile on the malleability of executive functions by tDCS and particularly highlight the task specificity of this interaction.

**FIGURE 1:**
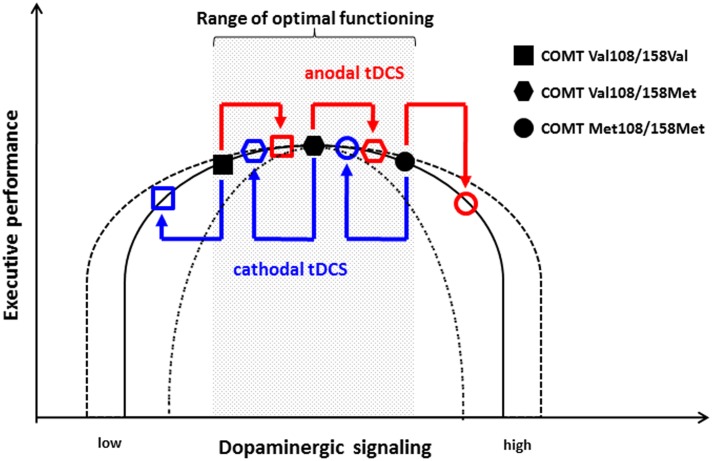
**Model of the effect of tDCS on the non-linear association between dopaminergic signaling and executive performance with regard to COMT Val108/158Met genotype.** Homozygous COMT Val108/158Val individuals are located on the ascending part of the curve. Therefore, anodal tDCS does not significantly impact their executive functioning, whereas cathodal stimulation causes deterioration. In contrast, homozygous COMT Met108/158Met individuals are located on the descending part. Consequently, their performance is not substantially affected by cathodal stimulation, whereas anodal stimulation leads to impaired cognitive functioning. According to this model, heterozygous COMT Val108/158Met individuals remain unaffected by both anodal and cathodal tDCS. The inverted-U shape curve might be highly variable between individuals and different tasks as indicated by the dashed line.

These results can be put in context of the inverted-U shape hypothesis in which both excessively high and low dopaminergic activity is associated with impairment (Schacht, 2016). Subjects homozygous for the Val allele have lower dopaminergic signaling and, therefore, are located more to the left on the inverted-U shape curve than homozygous Met allele carriers who have higher dopaminergic signaling. As **Figure 1** illustrates, this hypothesis suggests that the performance level of COMT Val108/158Val homozygous individuals is on the ascending side of the curve, whereas that of the Met108/158Met homozygous individuals is on the descending part. Based on this model, anodal tDCS might increase dopaminergic activity in Val108/158Val individuals in the range of optimal performance, which is why anodal stimulation does not have an effect on performance. In contrast, cathodal stimulation decreases the activity of dopaminergic neurons and shifts Val108/158Val individuals to lower performance levels. In turn, consistent with this model, the further increase of dopaminergic activity in Met108/158Met individuals by anodal stimulation leads to a deterioration of cognitive flexibility, as their dopaminergic tone is already relatively high. However, the cathodal decrease of excitability does not yield behavioral effects in these subjects with an already high dopaminergic activity.

Although these results fit well into this concept of an inverted-U shape relationship, many open questions remain. First, it will be necessary to disentangle the role of COMT Val108/158Met heterozygous individuals. In the two reported studies only individuals homozygous for either the Met or Val allele are significantly affected by anodal or cathodal stimulation, respectively. However, it is not clear if the stimulation actually has an intermediate effect on the heterozygous subjects. Second, it is of interest to further investigate the task specificity and state-dependency of the interaction between brain stimulation and genotype. The fact that in each study only one out of three executive functions showed a significant genotype-dependent modulation of performance is consistent with a differential influence of frontal dopamine concentrations on executive functioning. In this regard the influence of the COMT Val108/158Met polymorphism on changes in cognitive stability and flexibility after a tDCS-enhanced working memory training was recently investigated. However, no effects were found most likely due to a different study design targeting lasting transfer effects and/or a rather small sample size (Stephens and Berryhill, 2016). Another study related effects of tDCS over the right dlPFC on response inhibition to psychopathic traits like coldheartedness, since there is an association between psychopathic personality traits and impaired response inhibition. Here, a positive correlation between the score rating the participants’ coldheartedness and an improvement due to cathodal tDCS in their performance was found in the PGNG task measuring response inhibition (Weidacker et al., 2016). This is particularly remarkable in the context of the studies indicating an interaction between the COMT Val108/158Met polymorphism and tDCS (Plewnia et al., 2013; Nieratschker et al., 2015). Variability in executive functioning (Wishart et al., 2010) as well as antisocial behavior (Langley et al., 2010) have been linked with this gene. Therefore, the particular findings of this study might be based on a similar genetic profile particularly with respect to the COMT Val108/158Met polymorphism. However, the stimulation protocols used differed as Weidacker et al. (2016) applied stimulation to the right dlPFC before task completion, whereas Nieratschker et al. (2015) stimulated the left dlPFC during the task. Third, it will be important to also include genotype information from other polymorphisms. For instance, an interaction of the BDNF and the COMT polymorphisms has been demonstrated in a paired associative stimulation protocol inducing cortical plasticity (Witte et al., 2012). While no single polymorphism caused interindividual variability on its own it was shown that subjects homozygous for the BDNF Val allele, who were homozygous for the COMT Met allele at the same time, exhibited higher cortical plasticity. These results indicate a complex influence of the individual genetic makeup on the interaction between stimulation and cognition. Finally, epigenetic variability could also contribute to different tDCS responses. There is evidence from an animal study suggesting that long-lasting stimulation effects might be caused by epigenetic alterations of BDNF regulatory sequences increasing BDNF expression levels (Podda et al., 2016). Although epigenetic modifications are dynamic, certain baseline differences as well as variability in the epigenetic alterations potentially induced by tDCS could affect stimulation outcome.

To conclude, several studies indicate that genetic factors contribute to the interindividual variability of tDCS effects on cognition. Particularly, the COMT Val108/158Met polymorphism has been already demonstrated to shape the effects of tDCS on executive functions. Yet, the number of studies examining this interaction is still very small. Therefore, more research is needed to test the reliability of the existing data and to investigate the differential interactions of genetic disposition with specific cognitive processes and stimulation parameters. In addition, the complexity of this challenge is even increased by the critical interaction of different polymorphisms. However, for future brain-stimulation research the inclusion of genetic information in the design and analysis of brain stimulation studies, will essentially contribute to reduce the variability and allow for the development of more individualized stimulation protocols in basic and clinical research.

## Author Contributions

AW drafted the manuscript. VN and CP revised the article. All authors gave final approval of the version to be published.

## Conflict of Interest Statement

The authors declare that the research was conducted in the absence of any commercial or financial relationships that could be construed as a potential conflict of interest.
